# An eReferral Management & Triage System for minor Oral surgery referrals from primary care dentists: a cost-effectiveness evaluation

**DOI:** 10.1186/s12913-021-06775-9

**Published:** 2021-08-07

**Authors:** Harry Hill, Stephen Birch, Martin Tickle, Iain Petty, Joanna Goldthorpe

**Affiliations:** 1 School of Health and Related Research, University of Sheffield, Sheffield, UK; 2 Health Services Research & Primary Care, University of Manchester, Manchester, UK; 3 Centre for Business and Economics of Health, University of Queensland, St Lucia, Australia; 4 School of Dentistry, University of Manchester, Manchester, UK; 5 Division of Psychology & Mental Health, University of Manchester, Manchester, UK

## Abstract

**Objective:**

Oral surgery referrals from NHS dental practices are rising, increasing the pressures on available hospital resources. We assess if an electronic referral system with consultant or peer (general dental practitioner) led triage of patient referrals from general dental practices can effectively divert patients requiring minor oral surgery into specialist led primary care settings at a reduced cost whilst providing care of the same or enhanced quality. One year of no triage (all referrals treated in secondary care) was followed by one-year of consultant led triage, which in turn was followed by year of peer-led triage.

**Method:**

A health economic evaluation of all patient referrals from 27 UK dental practices for oral surgery procedures. The follow-up is over a three-year period at hospital dental services in two general hospitals, one dental hospital, and a single specialist oral surgeon based in two primary care practices. The evaluation is a comparison of mean outcomes in the hospitals and in specialist primary care dental services between the study periods (i.e. periods with and without the triage system). The main outcomes of interest are mean NHS cost saving per referral (costs to the NHS and costs to broader society), proportion of diverted referrals, case-mix of referrals and patient reports of the quality of dentistry services received at their referral destination.

**Results:**

The proportion of referrals diverted to specialist primary care was similar during both periods (45% under consultant-led triage and 43% under GDP-led triage). Statistically significant savings per referral diverted were found (£116.11 under consultant-led triage, £90.25 under GDP-led triage). There were no statistically significant changes in the case-mix of referrals. Cost savings varied according to the coding (and hence tariff) of referred cases by the provider hospitals. Patients reported similarly high levels of satisfaction scores for treatment in specialist primary care and secondary care settings.

**Conclusions:**

Implementation of electronic referral management in primary care, when combined with triage, led to appropriate diversions to specialist primary care. Although cost savings were realised by referral diversion these savings are dependent on the particular tariff allocation (coding) practices of provider hospitals.

**Supplementary Information:**

The online version contains supplementary material available at 10.1186/s12913-021-06775-9.

## Introduction

There has been a sustained increase in referrals from primary care dental practices into dental hospital services for dental surgery procedures, and it has been established that this contributes to significant pressures on National Health Service (NHS) finances due to higher costs in secondary care than in the primary care sector [1, 2]. An assessment of English oral surgery services, demonstrated that in a 3-year period from 2004, minor oral surgery referrals doubled from a monthly average of 182 to 364 [1]. This increase in referrals has been mirrored in medicine, and the factors that contribute to it include a lack of oral surgery experience at the undergraduate level among junior GDPs [3], and the increasing proportion of older patients retaining their teeth but presenting with complex medical histories [4].

A Cochrane systematic review identified three main approaches for effective management of the referral process to secondary care; professional education; referral management systems; and financial incentives to provide care within lower-priced specialist (or specialist led) primary care facilities, often referred to Tier 2 services [5]. Referrals require assessment to establish if the treatment needed is too complex for it to be delivered in lower cost Tier 2 services, which are services located in primary care. Therefore to ensure that such services are used appropriately some form of referral management is recommended by the NHS [6]. The NHS guidelines describe three main tiers of care that can be managed by referral systems in dentistry – Tier 1, standard primary care dental practice, Tier 2, intermediate or specialist centres and Tier 3, Consultant led services in secondary care. The system evaluated in this study is one that manages referrals to all three Tiers.

Oral surgery procedures are the most commonly referred by General Dental Practitioners (GDPs) to hospital services. Referral management systems frequently include a clinical triage stage where an appropriately trained clinician will view the referral and assess the appropriate Tier. In doing so, referrals are directed to the most appropriate service type. The purpose is to reduce waiting lists for surgery services in secondary care where capacity to treat patients is more limited than primary care and the cost of treatment is more expensive [7].

A dental care referral management system was implemented in 3 distinct phases. Previous to on-line referral management, referral letters from GDP were written directly to a named consultant and a dialogue was able to develop over treatment plans and individual cases. Phase 1 was the implementation of a passive on-line referral management and triage system without active redirection (all referrals were ultimately diverted to secondary care, regardless of the triager’s decision). A Consultant in oral surgery first examined all referrals that were supplied on an electronic form with an agreed minimum data set and referrals were categorised by the consultant into: suitable for secondary care services (Tier 3); suitable for primary care specialist services (Tier 2); suitable for any competent GDP to undertake (Tier 1); rejected due to insufficient information provided. All referred patients in this phase were treated in secondary care irrespective of the triage decision, but the numbers of cases appropriate for diversion to specialist primary care were recorded. The purpose of Phase 1 was a testing process to mitigate the risk of failure when “active” redirection of referrals takes place at the start of Phase 2. Using the same electronic referral system as Phase 1, the next two phases of the study used “active” triage to select a primary, specialist primary or secondary care provider. Phase 2 involved remote consultant-led decision on the triage destination of the patient (secondary or primary care). The consultants’ decisions are remote because the patient is not present and the triage decision is based entirely on the information entered onto the electronic referral system by the referring GDPs in primary care. In Phase 3, the referral form was modified to enable the referring GDPs (rather than a consultant working remotely from the referring primary care site) to select either a primary or secondary care destination for their referral. The decision to add GDP assessment of referral direction was based on the Kings Fund recommendation for peer assessment and the need to reflect local capacity for consultant triaging [8].

The first stage of the study took place in the calendar year 2014 (1 January through to 31 December 2014). Phase two and phase three took place in the calendar years of 2015 and 2016, respectively. Hence, data are collected at multiple and equally spaced time points (yearly), before (i.e. phase 1) and after interventions, with exact knowledge of when the interventions occurred: Jan 2015 for consultant led triage, and Jan 2016 for GDP led triage across all practices.

The aim of this study is to (1) estimate the impact of consultant-led and GDP-led active referral management and triage systems on the costs to the NHS and costs to broader society, (2) assess how the impact of the referral system on NHS costs differs by the provider of secondary care, and (3) assess patient satisfaction with the health care received from the provider of the minor oral surgery.

## Methods

### Population

The population were all referrals to secondary care in a large area in the North-West of England. The population area was chosen because it was a site with no previous referral management systems in place and referrals were made to 3 hospitals; two general hospitals accepting referrals for oral surgery and a dental hospital providing both service and training. All NHS dental practices in the region (*N* = 34) adopted the system. After excluding from the study practices that did not refer any patients, for example because they mainly use private oral surgeons who are not a part of the NHS system, the total number of practices available to contribute to the study was 27. Referrals could be directed for surgical treatment at one of the hospitals or from a specialist oral surgeon specialist operating at two primary dental service sites (further details of this specialist service can be found in a published case study) [9].

### Intervention

A primary dental care referral management system was implemented in 3 distinct phases, and overview of the interventions in place during these phases are summarised in Table 1. The new service began to refer patients to primary care at the start of Phase 2. The impact of Phase 2 and Phase 3 interventions were assessed by comparison with the period immediately prior to referral management being used (Phase 1).

**Table 1:** Implementation phases

	Year one (“Phase 1”): 2014	Year two (“Phase 2”): 2015	Year three (“Phase 3”): 2016
Study Phase	Virtual triage – health needs assessment	Full implementation -Electronic referral management with diversion to primary care or specialist primary care service	Full implementation with GDP autonomous decision making – whole system available with option of GDP triage
Impact and participant group most affected	Impact on primary care dental practices and commissioning through procurement of specialist primary care services. Consultants undertaking virtual triage.	Specialist primary care service provides some oral surgery and treatment outside of hospital settings; any impact on secondary care will start to be felt by hospital staff.	Autonomous GDP triage available with option remaining to refer triage activity to consultants. Commissioning staff look at rolling out the new system across a wider geographical footprint.

### Economic evaluation

The evaluation is a comparison of mean outcomes between study periods and takes a NHS perspective to costs and in a separate analysis a societal perspective. The outcome of the economic evaluation is cost per referral, specifically the difference in mean cost per referral avoided (i.e. redirected) to oral surgery services in different phases of implementation. Costs are from an NHS and societal perspective. The time horizon over which costs were compared was 2 years, with each year of “active management” compared to “no management”. Therefore, the mean cost per referral of “active” referral management programmes in Phase 2 and 3 will be compared to each other and in separate analysis each phase of “active management” will be compared to the year of no management (Phase 1). The e-referral data provides information on where the patient was referred to and final destination after their triage assessment. This allows to calculate the mean cost per referral in each hospital by restricting the sample of referrals compared across study phases to the subset of patients referred to the hospital.

Costs were assigned to all patients processed through the referral management system and treated in Hospital A (general hospital), Hospital B (a larger general hospital), Hospital C (a dental hospital) or primary care oral surgery services. All costs were based on 2015/2016 prices. We identified the costs for each oral surgery procedure in each of the three hospitals using Secondary Uses Data (SUD) covering the period 2013–2016 [10]. SUD is a comprehensive healthcare dataset in England that has treatment records form all patient contacts with the NHS. Each dental procedure was categorised into an outpatient, day case or elective (in-patient) stay and applied the corresponding national Tariff price (2015/2016). There were no changes to the tariff price for procedures over the study period. All NHS costs were inflated to 2019/2020 prices using the NHS cost inflation index [11].

The cost of referral is one element of total NHS cost but we also included the cost of any additional NHS care associated with complications that may have arisen. Information on the prevalence and type of treatment failures that had arisen during a one-month period post treatment was recorded in questionnaires completed by the patient (53.4% response rate). We coded the cost for the complications using the most frequently applied Tariff for that dental procedure in each of the hospitals (as revealed from the SUD).

Units of Dental Activity (UDAs) are the main currency of the 2006 primary care dental contract. The values of UDAs are specified in a contract agreement between the dentist and NHS England (typically around £25–30 per UDA) and the number of UDAs for each treatment course is specified nationally. An oral surgery procedure (tooth extraction) attracts a Band II tariff of 3 UDAs. Tier 2 primary care services will attract a higher UDA rate to reflect the additional complexity and hence a fixed tariff of £150 reflecting a £50 UDA rate was used. As all procedures in specialist primary care was at a fixed tariff, cost savings per referral reported represent the difference in surgical procedure cost between the primary and secondary care sectors. The cost of the referral management programme was £8.22 per consultant referral (Phase 2) and £4.00 per GDP referral (Phase 3). This cost was based on the cost of the referral management infrastructure (i.e. the fixed cost to build and maintain the platform) and a fee for the consultant to triage. GDP referral costs are included within the fee paid to assess and treat an attending patient.

In addition to the cost to the NHS we estimated the costs associated with time travelling to and from an appointment and the personal costs of travel to provide an estimate of patient/family costs which, when added to costs to the NHS represent total societal costs. The unit costs for indirect costs are summarised in the Additional file 1. Costs to patients and patient carers (i.e. accompanying persons to the appointment) were calculated from questionnaire data completed by referred patients on the costs incurred in travelling to and from, waiting and being treated at the healthcare facilities of their triage destination. These costs included NHS prescription items (painkillers or antibiotics), the cost of lost time as well as out-of-pocket costs for public or private transport, parking etc.

### Case-mix

All referrals were in Phase 2 and Phase 3 were assessed had the following case-mix metrics recorded for the purpose of indicating treatment complexity: age, American Society of Anesthesiologists status, smoking status, alcohol status. This data is used to test case-mix changes between phases.

### Quality of dentistry service

Patients referred through the system in Phase 2 and 3 were approached to see if they would consent to complete a questionnaire to provide their views of the service. Of 1427 patients invited to participate via a letter sent through the post, 402 (28.2%) patients consented to complete the questionnaire following their surgical procedure. A total of 214 (53.2% of consenting patients) completed and returned questionnaires (postal and online questionnaire options were given).

## Results

### Referral volumes and case-mix

The referral volumes and the final provider destinations are shown in Table 2. The total number of referrals in Phase 2 was 643, with 45% diverted by Consultants to primary care services, 53% received in secondary care and 2% sent back to the original GDP referrer. In Phase 3 a total of 861 referrals for oral surgery were sent with 43% diverted to primary care services by the referring GDP.

**Table 2:** Redirected referrals to primary and secondary care

Triage Type	Phase 1 (2014)		Phase 2 (2015)		Phase 3 (2016)	
	No active triage		Consultant		GDP	
	N	%	N	%	N	%
Redirected to primary care	0	0	15	2	0	0
Redirected to specialist primary care oral surgery service	0	0	287	45	369	43
Treated in secondary care by consultants	670	100	341	53	492	57
**Total**	**670**	**100**	**643**	**100**	**861**	**100**

In Phase 1 (no triage system in place) all patients referred to secondary care were treated in secondary care. This was reduced to 53% of all referrals in Phase 2 (consultant-led triage) and 57% of all referrals in Phase 3 (GDP-led triage). Thus, patients were more likely to be redirected to specialist primary care services under consultant led triage although the difference between the two active phases was small. Only 5 referrals across both study phases were subsequently regarded by the dentists in specialist primary care as inappropriate for treatment in primary care (i.e. referral more complex than anticipated) and sent back. There was no significant difference on any of the metrics assessing case-mix between primary and secondary care settings, and between Phase 2 and Phase 3.

### Quality of care received

We found no statically significant difference at a 5% level in the prevalence of treatment failures (complications, urgent care post-surgery) between study phases and between primary and secondary care. Table 3 presents aggregated satisfaction questionnaire data for participants with a redirected referral in Phases 2 and 3. These data were not used to compare patient responses for Phases 2 and 3, due to the relatively small numbers, the possibility of non-response bias and lack of variation in in the answers. Above 90% report being satisfied with their dentist and above 80% would recommend treatment at the same place to someone with a similar dental complaint. However, these results are descriptive. The poor response rate means that we are unable to draw any inferences relating to quality of care.

**Table 3:** Quantitative results from the patient satisfaction questionnaire

Answers	Was this the surgery/hospital you wanted?		Were you satisfied with your dentist’s explanation of why you were being referred for oral surgery?		Did the surgery resolve/fix your dental problem?		Would you recommend treatment at the same place to someone with a similar dental complaint?		Have you had to return to the surgeon or your own dentist for any complications due to your procedure?	
	PC%	SC%	PC%	SC%	PC%	SC%	PC%	SC%	PC%	SC%
No	9	4	4	5	5	16	5	3	84	79
Yes	39	60	95	92	85	57	82	93	16	21
Don’t Mind	53	36	0	0	0	0	0	0	0	0
Not sure	0	0	1	3	10	27	13	4	0	0
*P* value	0.01		0.48		< 0.001		0.07		0.45	

*PC* primary care, *SC* secondary care

### Costs

Table 4 shows the number of procedures at each hospital categorised into outpatient, day case or elective (in-patient) procedures. The coding profile demonstrates considerable differences between hospitals in the allocation of the minor oral surgery referrals to outpatient care, day case or elective (inpatient) categories. There are clear differences in the way that similar procedures were coded in each hospital. For example, in Hospital A, many procedures were coded as day case, while in Hospital C, there was very little use of the day case tariff and much greater use of the (lower) outpatient tariff.

**Table 4:** Number of outpatient tariffs for top five procedures per hospital

Hospital Code & Type	Procedure description	Outpatient tariff	Day Case tariff	Elective
**Hospital A** **General hospital**	Surgical removal of wisdom tooth NEC	0	444	1
	Surgical removal of retained root of tooth	0	430	4
	Unspecified simple extraction of tooth	8	259	1
	Apicectomy of tooth	0	206	0
	Surgical removal of tooth NEC	0	200	1
**Hospital B** **Large general hospital**	Unspecified simple extraction of tooth	1054	94	3
	Biopsy of lesion of mouth NEC	781	42	12
	Excision of lesion of mouth NEC	479	44	10
	Surgical removal of retained root of tooth	385	87	16
	Surgical removal of wisdom tooth NEC	326	777	57
**Hospital C** **Dental hospital**	Unspecified simple extraction of tooth	742	25	0
	Surgical removal of tooth NEC	162	15	0
	Surgical removal of wisdom tooth NEC	111	29	0
	Surgical removal of impacted wisdom tooth	72	25	0
	Surgical removal of retained root of tooth	67	4	0

### Health economics

The difference in referral costs for each hospital, for each phase of the study are shown in Table 5 and the top five ranking procedures, which were almost entirely tooth extractions. This shows the change in costs between study phases divided by the change in total number of all referrals (not just the diverted referrals). The mean NHS cost saving per referral under consultant-led triage (Phase 2) compared to no active triage (Phase 1) was £116.11. After including patient costs, the mean societal cost saving per referral was £111.49. The mean NHS and societal cost savings per referral from GDP-led triage (Phase 3) compared to no active referral (Phase 1) were smaller, £90.25 and £86.12 respectively. Each of these cost differences are statistically significant (*P* < 0.01). The mean societal cost savings per referral for Hospital A and Hospital B under consultant-led triage were £164.44 and £135.04 respectively, and with GDP-led triage these decreased to £150.02 and £93.52. In Hospital C there was no statistically significant change in the mean cost per referral between study phases. The average cost of complications per referral in primary was less than in secondary care, at £0.62 and £3.28 respectively, due to the lower number of complications in primary care (Table 3).

**Table 5:** Mean cost difference per referral avoided (compared to Phase 1) by hospital provider

Implementation phases compared in the evaluation	Secondary care provider used in the evaluation	NHS cost difference per referral (standard error), ***p*** value	Societal cost difference per referral (standard error), ***p*** value
**Phase 1 vs** **Phase 2**	All secondary care	**- £116.11** (£12.43), *p <* 0.01	**- £111.49** (£12.41), *p <* 0.01
	Hospital A only	**- £169.34** (£12.95), *p <* 0.01	**- £164.44** (£12.78), *p <* 0.01
	Hospital B only	**- £135.93** (£33.93), *p <* 0.01	**- £135.04** (£33.91), *p <* 0.01
	Hospital C only	£0.90 (£7.19), *p* = 0.90	£10.00 (£7.27), *p* = 0.17
**Phase 1 vs** **Phase 3**	All secondary Care	**- £90.25** (£12.40), *p <* 0.01	**- £86.12** (£12.39), *p <* 0.01
	Hospital A only	**- £154.54** (£13.17), *p <* 0.01	**- £150.02** (£13.02), *p* < 0.01
	Hospital B only	**- £93.90** (£31.89), *p <* 0.01	**- £93.52** (£31.87), *p <* 0.01
	Hospital C only	£12.02 (£6.69), *p* = 0.07	£14.30 (£6.82), *p* = 0.06

Societal cost savings were less than NHS cost savings because the mean time and travel costs to primary care (dental practices and the one specialist primary care facility) were higher than the travel and time costs to secondary care services which were delivered at one of three different locations.

## Discussion

The referral management programme generated savings in both Phase 2 and Phase 3 when compared to no system in place under Phase 1. This was the case when the total costs of the referral management system, primary and specialist primary care services and all hospital systems are considered. The savings were higher for referrals diverted from Hospital A, while there were no statistically significant differences in costs between no referral management and the referral management phases where referrals were diverted from Hospital C. These savings were generated from differences in costs in extraction procedures between secondary and primary sectors. The absence of savings in Hospital C is due to the hospital’s frequent use of an outpatient tariff rather than the costlier inpatient tariff which resulted in procedure costs that were almost identical to specialist primary care.

We found a very high level of satisfaction with the health care received from patients in the redirected referral system. Although this presumably would not be the case if the system had caused serious harm or inconvenience to patients, drawing conclusions on the quality of service is difficult due to the low response rate of 15% and because there was no specified time period for which the patients were asked to return the satisfaction questionnaire. Consequently, post-surgical follow-up duration was not known and not uniform: high satisfaction may represent early responses, those questionnaires returned within a few days of surgery before an event of post-surgical complication had occurred. A separate analysis has however found high patient adherence in the system, which presumably would not have been the case if patients viewed their triage destination negatively. The analysis showed that a majority of patients (87%) that were redirected to have surgery at a primary care specialist service attended their appointment. A nested qualitative study found that patients’ priorities were largely indifferent to how services were organised and the location they were ultimately treated under [12].

The main strength of the study is the use of a real-world setting. There was no formal referral management in place and all NHS dental practices in the region adopted the system at the same time. This removed any potential selection bias of dental practices into this study. The availability of three large hospitals serving the same local area enhances the generalisability of our results to other areas. The use of hospital specific tariff applications in the health economic evaluation permitted a more sophisticated understanding of the impacts of referral diversions than could have been achieved by costing hospital procures using national average procedure costs or tariff guidance costs.

The evaluation would have benefited from a comparison of cost changes under the referral management programme to changes that may have occurred in the oral health gains from treatment but this was beyond the scope of the current study. A limitation was the relatively small geographical footprint of the study setting. This meant the alternative to receiving surgery treatment in secondary care was from a single specialist primary care surgeon operating at two primary care sites. Therefore the estimates of societal costs, specifically the costs to patients of travelling to and from their surgery appointment, is highly influenced by the location of these two particular practices. The referral population may also not represent the rest of the United Kingdom (UK), in terms of the proportion of referrals that are assessed in triage to be suitable for treatment in primary care, specialist primary care or secondary care. Therefore, the impact of referral management systems needs to be assessed in different geographical, social and service contexts. It is particularly important to assess the impact of any new NHS dental contract on the volume and appropriateness of referrals to specialist services. Finally, we had no a control group i.e. a region where no e-referral system was implemented. Therefore we cannot say with certainty that the changes we observed would not have happened without the intervention.

The total savings from the use of consultant triage amounts to £40,189 (details provided in the Additional file 1). There are other benefits to the triage beyond the cost saving and patient satisfaction benefits discovered in this study. It is possible that, by freeing up capacity at hospitals, the use of the e-referral system and triage may have reduced waiting times. There is evidence from interviews with referral patients in this triage system that some had perceived shorter waiting times and more choice in out of hours’ appointment times [9]. The system also provides transparent information on the flows of referrals through the healthcare system. This up-to-date information could help commissioners actively manage the commissioning process in a flexible way. There is also evidence in the research literature to suggest consultant triage may improve quality and appropriateness of dental referrals. A recent review focussed on the effectiveness and efficiency of moving hospital services (outpatients) into primary care and examined 184 studies, some of which included dental settings [13]. Their conclusions, that minor surgical procedures can be carried out in primary care safely and effectively and the cost effectiveness of these services depending on local conditions, coincides with our findings even though our setting (minor oral surgery) is different.

## Conclusion

The combination of a referral management system and primary care oral surgery service offers potential significant cost savings to the NHS. The effect on costs was context-specific, and, depending on the coding behaviour of the different hospitals, showed large variation. There is a need to ensure consistent, and accurate coding is essential if cost savings from referral management are not to be overestimated. A realistic estimate of cost savings requires an extensive and robust evaluation of the local tariff costs but any changes could potentially result in a loss of resources for NHS England commissioning groups and could destabilise service delivery. Nonetheless this study shows the importance of understanding the tariff landscape prior to embarking on implementation of a referral management system, if the established cost savings are to be sustainable and meaningful. Irrespective of the impact on NHS costs, we found referral management reduced demands on secondary care facilities and released capacity for reducing wait times for secondary care.

## Supplementary Information

**Additional file 1.**

## Data Availability

The datasets used and/or analysed during the current study available from the corresponding author on reasonable request.

## Abbreviations

GDP: General dental practitioners
NHS: National Health Service
SUD: Secondary Uses Data
UDA: Units of Dental Activity
UK: United Kingdom
