# Supplementary material for: An eReferral Management & Triage System for minor Oral surgery referrals from primary care dentists: a cost-effectiveness evaluation
Source: BMC Health Serv Res. 2021 Aug 7;21:781. doi: 10.1186/s12913-021-06775-9 (PMC8349481; doi:10.1186/s12913-021-06775-9)
Supplement: Supplementary file 1 — Additional file 1. [file 12913_2021_6775_MOESM1_ESM.docx]

**Additional file 1**

**Supplementary Table 1** Unit costs for indirect costs.

| **Indirect cost** | **Cost** | **Reference for price of indirect cost** |
| --- | --- | --- |
| Travel cost – Car | £0.45 per mile | HMRC tax rates per business mile (First 10,000 miles)^1^ |
| Travel cost - Motorbike | £0.24 per mile | HMRC tax rates per business mile (First 10,000 miles)^1^ |
| Travel cost – Cycle | £0.20 per mile | HMRC tax rates per business mile (First 10,000 miles) ^1^ |
| Travel cost – Train | £0.30 per mile | Based on Macclesfield to Manchester trip. |
| Travel cost – Taxi | 1 mile: £3.90  3 miles: £7.50  5 miles: £11.20  7 miles: £15.00  9 miles: £18.60  10 miles: £20.40 | Manchester local government authority. [Hackney Carriage Fare Review 2016.](https://www.westsuffolk.gov.uk/Council/Consultations/hackneycarriagefarereview2018stage2publicconsultation.cfm) |
| Travel cost – Bus | £0.45 per mile | Average (in sample) ticket price |
| Travel cost – Walk | - | - |
| Parking tickets and other ticketed travel expenditures | Reported by the patient | Average (in sample) ticket price |
| Cost of travel time to and from an appointment | £15.73 per hour | Office for National Statistics^2^ |
| NHS prescription items (due to complications) | £8.40 per item | NHS Choice website |

**References:**

1. Travel - mileage and fuel rates and allowances. Available at: <https://www.gov.uk/government/publications/rates-and-allowances-travel-mileage-and-fuel-allowances/travel-mileage-and-fuel-rates-and-allowances>
2. Office for National Statistics. Earnings and working hours 2016. Available at: <https://www.ons.gov.uk/employmentandlabourmarket/peopleinwork/earningsandworkinghours>

**Total savings resulting from the use of consultant triage**

Supplementary Table 2 reports the distribution of referrals together with the relative management costs for those referrals where there was a listed decision by both groups. These were then applied to mean costs in secondary care with the resultant cost differences shown in Supplementary Table 3. The total savings from the use of consultant triage amounts to £40,189.

Supplementary Table 2**:** Decisions made by GDPs and consultants in phase 3

| Triage type | Care setting, number of decisions | | |
| --- | --- | --- | --- |
|  | Primary (Level 2) | Secondary (Level 3) | Triage cost (£) |
| GDP | 251 | 581 | 4.00 × 832 = 3328 |
| Consultant | 384 | 412 | 8.22 × 796 = 6543 |

Supplementary Table 3**:** Cost differences when comparing GDP with consultant triage in phase 3 (NHS Costs)

| Triage type | Care setting, number of decisions | | | Cost (£) | | | |
| --- | --- | --- | --- | --- | --- | --- | --- |
|  | Primary (Level 2) | Secondary (Level 3) | Triage cost (£) | Primary | Secondary | Triage cost (£) | Total |
| GDP | 251 | 581 | 4.00 × 832 = 3328 | 179.74 × 251  = 45,115 | 401.81 × 581  = 233,452 | 4.00 × 832  = 3328 | 45,115 + 233,452 +  3328 = 281,895 |
| Consultant | 384 | 412 | 8.22 × 796 = 6543 | 179.74 × 384  = 69,020 | 401.81 × 412  = 165,546 | 8.22 × 796  = 6543 | 69,020 + 165,546 +  6543 = 241,109 |
| Difference |  |  |  |  |  |  | 40,189 |
